# Malignant lymphomas (ML) and HIV infection in Tanzania

**DOI:** 10.1186/1756-9966-27-9

**Published:** 2008-06-10

**Authors:** Amos R Mwakigonja, Ephata E Kaaya, Edward M Mgaya

**Affiliations:** 1 Department of Pathology, Muhimbili University of Health and Allied Sciences (MUHAS), Tanzania; 2 Immunopathology Lab. R8:00, Cancer Center Karolinska (CCK), Department of Oncology-Pathology, Karolinska University Hospital Solna/Karolinska Institute, Stockholm, Sweden

## Abstract

**Background:**

HIV infection is reported to be associated with some malignant lymphomas (ML) so called AIDS-related lymphomas (ARL), with an aggressive behavior and poor prognosis. The ML frequency, pathogenicity, clinical patterns and possible association with AIDS in Tanzania, are not well documented impeding the development of preventive and therapeutic strategies.

**Methods:**

Sections of 176 archival formalin-fixed paraffin-embedded biopsies of ML patients at Muhimbili National Hospital (MNH)/Muhimbili University of Health and Allied Sciences (MUHAS), Tanzania from 1996–2001 were stained for hematoxylin and eosin and selected (70) cases for expression of pan-leucocytic (CD45), B-cell (CD20), T-cell (CD3), Hodgkin/RS cell (CD30), histiocyte (CD68) and proliferation (Ki-67) antigen markers. Corresponding clinical records were also evaluated. Available sera from 38 ML patients were screened (ELISA) for HIV antibodies.

**Results:**

The proportion of ML out of all diagnosed tumors at MNH during the 6 year period was 4.2% (176/4200) comprising 77.84% non-Hodgkin (NHL) including 19.32% Burkitt's (BL) and 22.16% Hodgkin's disease (HD). The ML tumors frequency increased from 0.42% (1997) to 0.70% (2001) and 23.7% of tested sera from these patients were HIV positive. The mean age for all ML was 30, age-range 3–91 and peak age was 1–20 years. The male:female ratio was 1.8:1. Supra-diaphragmatic presentation was commonest and histological sub-types were mostly aggressive B-cell lymphomas however, no clear cases of primary effusion lymphoma (PEL) and primary central nervous system lymphoma (PCNSL) were diagnosed.

**Conclusion:**

Malignant lymphomas apparently, increased significantly among diagnosed tumors at MNH between 1996 and 2001, predominantly among the young, HIV infected and AIDS patients. The frequent aggressive clinical and histological presentation as well as the dominant B-immunophenotype and the HIV serology indicate a pathogenic association with AIDS. Therefore, routine HIV screening of all malignant lymphoma patients at MNH is necessary to enable comprehensive ARL diagnosis and formulation of preventive and therapeutic protocols.

## Background

After a long asymptomatic period of variable duration, HIV infection results in lowered immunity with increased susceptibility to new and/or normally latent infections and opportunistic tumors characteristic for the late stage called acquired immune deficiency syndrome (AIDS). Depending on the region, 25–40% of HIV-1 seropositive patients eventually develop a malignancy predominantly Kaposi's sarcoma (KS) and malignant lymphoma (ML) mostly sub-classified as non-Hodgkin (NHL) [including Burkitt's lymphoma (BL)] and Hodgkin's lymphoma (HL) also known as Hodgkin's disease (HD) which were shown to increase with the HIV epidemic in the USA. [1,2] In Tanzania a higher proportion of NHL has been reported [3] during the AIDS epidemic as shown by a frequency of 9.1% in males in Dar es Salaam (1990–91) compared to the 6.3% during 1980–81 pre-AIDS period. [4] Corresponding figures for females during the same time periods were 3.5%. Reported data from Uganda showed an increase in age standardised rates (ASR) for NHL for the periods 1954–60 and 1993–97 of 3.2 to 5.7 for males and 2.9 to 4.2 per 100,000 for females. [3] Rare ML subtypes namely primary effusion lymphoma (PEL) or body cavity-based lymphoma (BCBL) as well as primary central nervous system lymphoma (PCNSL) are also more often associated with AIDS. [5,6] ML occurring in HIV infected persons are called AIDS-related lymphomas (ARL) and have distinct clinical and pathogenetic characteristics including, extra-nodal presentation, systemic dissemination, B-cell phenotype, presence of Epstein-Barr virus (EBV) markers in tumor cells and recurrent genetic lesions. [6-8] About 70–90% of ARL are predominantly high-grade diffuse large cell (DLBCL) having poor prognosis. [9] A similar entity has also been described in experimental simian AIDS-related lymphomas (SARL). [7]

Although it is not regarded as an AIDS-defining malignancy, HD appears also to occur more commonly in association with AIDS particularly the mixed cellularity (MC) and lymphocyte depletion (LD) subtypes [10-13] and to have clinico-biological features similar to those of AIDS-related NHL. [6,11] It appears that HIV infection alters the clinical course of HD and therefore advanced or high-grade HD in HIV-infected individuals should be considered indicative of AIDS. [13]

Burkitt's lymphoma, first described in Africa by Dennis Burkitt in 1958, is a B-cell neoplasm and the commonest childhood tumor in equatorial Africa. [14,15] The incidence of BL in North Mara, Tanzania from 1964 to 1970, was 5.7/100,000 being higher in males than in females. [16] An incidence of BL in Kilimanjaro (high altitude) region was found to be 1.4/100,000 for boys and 0.7/100,000 for girls aged 5–9 years for the period 1971–1980. [17] This study revealed geographical variations that were related to altitude as was also shown by the proportional ratios for BL of 11.8% of tumours in North Mara [18] and of 21.6% in males in Tanga (both low altitude areas). [19]

Surprisingly however, an increased incidence of ARL has so far not been consistently observed in other regions of Africa. However, the association of NHL, HD and BL with HIV and AIDS generally in Africa including Tanzania is poorly documented and needs further clarification. [20,21]

## Methods

### Study area

The equatorial East African country of Tanzania has a population of approximately 35 million with roughly a 1:1 male: female ratio (National Bureau of Statistics, 2002).

### HIV serology

Evaluation of HIV-1 infection (by ELISA) was performed at the Microbiology/Immunology department at Muhimbili University of Health and Allied Sciences (MUHAS) as previously described. [22]

### Biopsies

Archival formalin-fixed paraffin-embedded (FFPE) diagnostic ML tissue biopsies (176) delivered to the Histopathology Laboratory, Muhimbili National Hospital (MNH), Dar es Salaam from 1996 to 2001 were reassessed and selected (based on quality and amount of tissue material/block) ML biopsies (70) were immunoassayed. Corresponding clinical files and histological reports were also retrieved.

### Histology and immunohistochemistry (IHC)

This was done at the Department of Histopathology and Morbid Anatomy (MNH/MUHAS). Briefly, tissue sections (5 μ thick) were stained with Hematoxylin and Eosin (H & E) as previously described.[12,22]

For IHC the avidin-biotin complex (ABC) immunoperoxidase technique was used as previously described [12,22] on tissue sections, mounted on SuperFrost^® ^slides (Menzel GmbH & Co KG, Braunschweigh, Germany) deparaffinized, rehydrated and boiled in a microwave for 6 minutes in citrate buffer at pH 6 and 750W for antigen retrieval. Quenching of endogenous peroxidase activity, was done by incubating the sections in 30% hydrogen peroxide in distilled water for 30 minutes at room temperature, washing in Tris-buffered saline (TBS) followed by incubating with 1:20 normal serum from the species providing the secondary antibody and washed (TBS). The sections were then incubated overnight at 4°C with the respective primary antibody (diluted 1:50) including respectively, mouse monoclonal to pan-leucocyte anti-human CD45 (clone LCA), B-cell anti-human CD20 (clone L26), macrophage anti-human CD68 (clone Kp-1), the anti-human Ki-67 (MIB-1) proliferation marker or the rabbit polyclonal anti-human CD3 (T-cell) marker all anti-mouse/rabbit antibodies from DakoCytomation, (Glostrup, Denmark). Subsequently, the sections were rinsed with buffer and incubated with ABC, washed and developed (visualized) with Sigma DAB chromogen (Sigma-Aldrich, St. Louis MO, USA) as previously described [12,22] and after TBS washing, lightly counter-stained with Harris Hematoxylin, blued in running tap water for 30 minutes, dehydrated in ascending grades of ethanol, cleared in two runs of xylene and mounted with DPX and coverslipped.

Negative controls included sections from tissues not expressing the respective antigen as well as substitution of the primary antibody by buffer. Positive controls included tissue sections known to express the antigen under investigation.

### Microscopic evaluation

Sections were analyzed using a Carl Zeiss axiomatic photomicroscope (Jena, Germany). Cell reactivity was expressed as mean positive cells/total number of cells in 8 high (× 400) power fields (HPF). Picture processing and printing was done using Adobe Photoshop 7.0 (Adobe Systems Incorporated, San Jose, USA) and Microsoft-Power Point 2003 (Microsoft Corporation, Redmond, WA, USA).

### Statistical analysis

The coded results were analyzed in a PC computer using the EPI INFO 6 statistical software (CDC, Atlanta, Georgia, USA). P-values of ≥ 0.05 were considered statistically significant.

### Ethical considerations

A strictly confidential specimen processing and evaluation was conducted. HIV screening was performed upon informed consent in the respective wards and clinics at MNH and the study was conducted after appropriate review by MUHAS Institutional Review Board (IRB) and Ethical Clearance, under the TANSWED HIV Project E.

## Results

### General demography and ML frequency

**Figure 1:**
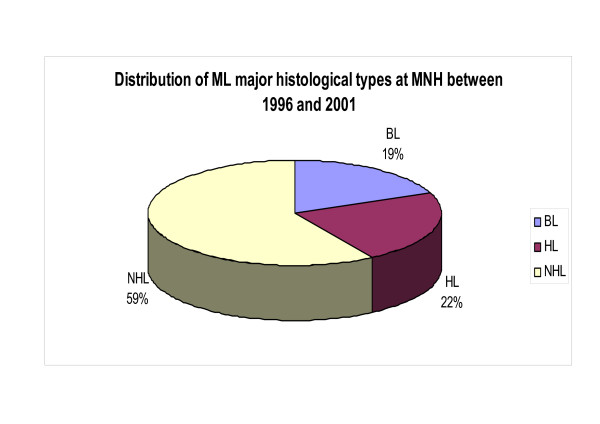
Pie chart showing the proportion of different ML types at MNH between 1996 and 2001. Note that NHL formed the largest group.

**Figure 2:**
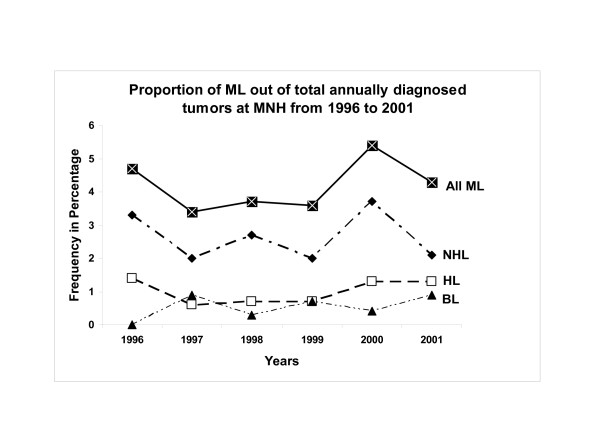
Frequency polygon showing variation of different ML types with time at MNH between 1996 and 2001. Note the increase in frequency of NHL and HD between 1997 and 2000.

Out of a total of 4200 diagnosed tumors 176 ML (4.2%) were consecutively collected at MNH during the 1996–2001 period. Of these, 112 (63.6%) were males and 64 (36.4%) females corresponding to a male:female (M:F) ratio of 1.8:1 (Table 1). Age data was available for 171 cases and the mean and median ages at diagnosis for those ML were 30.23 and 25.00 years respectively (standard deviation [SD] = 21.45) and the age range was 3–91 years, (Table 1). Of the total ML, 59 were NHL, 19.0% BL (34) and 22.0% HD (39) [Figure 1]. It appears that ML increased from about 0.42% in 1997 to 0.70% in 2001 largely due to cases of NHL (2.0–3.7%) and HD (0.6–1.3%) whereas the incidence of BL remained constant, which differences were statistically highly significant (p-value = 0.001) [Figure 2].

**Table 1:** Frequency of ML at MNH by age, sex and HIV serostatus

DIAGNOSIS	AGE (Years)			SEX [NO (%)]	HIV SEROSTATUS [NO (%)]				TOTAL
	**MIN**	**MAX**	**MEAN**	**M:F Ratio**	**Positive**	**Negative**	**Tested**	**Not-tested**	**NO (%)**
	
**NHL**	4	91	39.01	66(64.1)/37(35.9) = .1.8:1	5 (25.0)	15 (75.0)	**20 (19.4)**	**83 (80.6)**	**103 (58.52)**
**HD**	5	70	26.42	25(64.1)/14(35.9) = **1.8:1**	4 (28.6)	10 (71.4)	**14 (35.9)**	**25 (64.1)**	**39 (22.16)**
**BL**	3	20	8.94	21(61.8)/13(38.2) = **1.6:1**	0 (0.0)	4 (100.0)	**4 (11.8)**	**30 (88.2)**	**34 (19.32)**

**NHL+HD+BL**	**4**	**91**	**30.23**	**112(63.6)/64(36.4) = 1.8:1**	**9 (23.7)**	**29 (76.3)**	**38 (21.6)**	**138 (78.4)**	**176 (100.0)**

### Non-Hodgkin's lymphoma (NHL)

**Figure 3:**
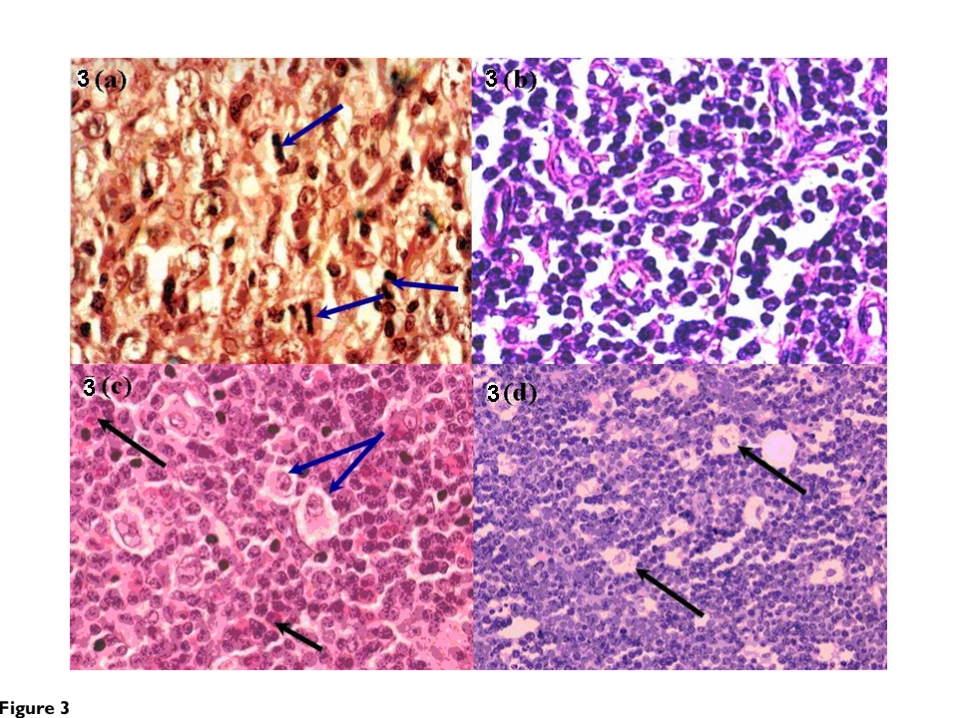
**(a) **H & E section of a diffuse large cell lymphoma (DLBCL) [high grade NHL]; note the high mitotic index [blue arrows] (× 400). **(b) **H & E section of a small cell lymphoma [SCL] which is a low-grade NHL (× 400). **(c) **H & E section of a Hodgkin's disease mixed cellularity (HDMC) case; note the classical Reed-Sternberg (R-S) cell [blue arrows] and eosinophil cells [black arrows] (× 400). **(d) **H & E section of a BL; note the "starry-sky" appearance due to tingible-body macrophages [black arrows] (× 200).

The age range for NHL was 4–91 years with a mean and median age of 42 and 39.01 years respectively (Table 1) and more males (64.08%) were affected by NHL (66/103) than females (35.92%, 37/103) but the distribution of NHL histological subtypes with sex was not significantly different (p = 0.23) [Table 2]. High-grade NHL [Figure 3(a)] accounted for 62.14% (64/103) of total NHL while low-grade histological types [Figure 3(b)] accounted for 37.86% (39/103) [Table 2]. The age ranges for high-grade NHL (HGNHL) and low-grade NHL (LGNHL) were 14–76 and 4–91 years and the mean age 36.06 and 44.11 years respectively, which differences were highly statistically significant (p-value = 0.0036) [Table 2].

**Table 2:** Distribution of ML histological subtypes by sex and age

HISTOLOGY	MEAN AGE (Years)	SEX [NO (%)]		TOTAL
**NHL**		**Male/Female**	**M:F Ratio**	
LGNHL	44.1	30(76.9)/9(23.1)	3.3:1	**39 (23.4)**
HGNHL	36.0	37(57.8)/27(42.2)	1.4:1	**64 (70.3)**

**TOTAL NHL**		**67(65.1)/36(34.9)**	**1.9:1**	**103 (100.0)**

**HD**				
LP	34.2	3(50.0)/3(50.0)	1:1	**6 (15.4)**
LD	70.0	0/1(100.0)	N/A	**1 (2.56)**
MC	24.3	12(60.0)/8(40.0)	1.5:1	**20 (51.3)**
NS	23.0	8(80.0)/2(20.0)	4:1	**10 (25.6)**
NOS*		2(100.0)/0	N/A	**2 (5.1)**

**TOTAL HD**		**25(64.3)/14(35.7)**	**1.8:1**	**39 (100.0)**

*Not **O**therwise **S**pecified

### Hodgkin's disease (HD)

Over half of the HD cases (51.30%, 20/39) were of mixed cellularity (MC) histology [Figure 3(c)] followed by the nodular sclerosing (NS) (25.6%, n = 10/39) and lymphocyte predominance (LP) (15.4%, n = 6/39) subtypes. There was only one case of lymphocyte depletion (LD) subtype in the material (Table 2). The age range for HD was 5–70 and the mean age 26.42 years (Table 1). The age ranges for HD according to subtypes were 11–51, 7–57, and 5–41 years for LP, MC and NS respectively. The mean ages were 34.20, 24.32 and 23.00 years for LP, MC and NS respectively and the patient with LD was 70 years old (Table 2).

### Burkitt's lymphoma (BL)

The age range for BL cases [Figure 3(d)] was 3–20 years and the mean and median ages were 8.94 and 7.00 years respectively (Table 1). A male preponderance (61.8%, 21/34) for BL over females (38.2%, 13/34) was highly statistically significant (p-value < 0.0001) (Table 1).

### HIV association of lymphomas

**Figure 4:**
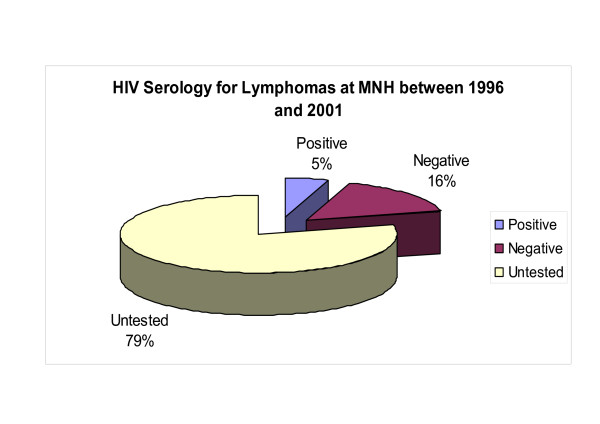
Pie chart showing the proportion of ML serologically screened for HIV antibodies at MNH between 1996 and 2001. Note that a large number of ML at MNH were not screened for HIV.

Only 21.6% (38/176) of the ML patients were available for testing by serology for HIV infection during the study period and nine (23.7%) tested positive including five (55.6%) NHL (all B-cell type), and four (44.4%) HD while all screened BL (4) were seronegative (Table 1 and Figure 4). Most seropositive NHL (80%, n = 4/5) were high-grade and of B-cell phenotype (see below) although this was not statistically significant (2-tailed p-value = 0.6126, Fisher Exact test) [Table 3]. No correlation of HD histological subtypes with HIV was statistically significant (Table 1).

**Table 3:** Distribution of HIV antibody serostatus by histological grade of NHL at MNH (1996–2001)

	GRADE		
		
HIV SEROSTATUS	LGNHL	HGNHL	TOTAL NO (%)
**POSITIVE**	1 (20.0%)	4 (80.0%)	5 (29.4%)
**NEGATIVE**	6 (40.0%)	9 (60.0%)	15 (70.6%)

**TOTAL**	**7 (35.0%)**	**13 (65.0%)**	**20 (100.0)**

2-Tailed p-value = 0.6126 (Fisher Exact Test)

### Clinical presentation

Most (65.3%) ML patients (115/176) had localized tumors but disease dissemination increased significantly with age, p-value = 0.038. More (58.5%) patients (n = 103/176) presented primarily with supra-diaphragmatic than sub-diaphragmatic ML which difference was statistically highly significant (p-value < 0.0001). Most of the HIV seropositive ML (66.7%, n = 6/9) had generalized disease (χ^2 ^= 21.06, p-value = 0.00031) and either isolated supra-diaphragmatic (66.7%, n = 6/9) or bi-diaphragmatic but not isolated sub-diaphragmatic disease (p-value = 0.00039).

### Immunohistochemistry

**Figure 5:**
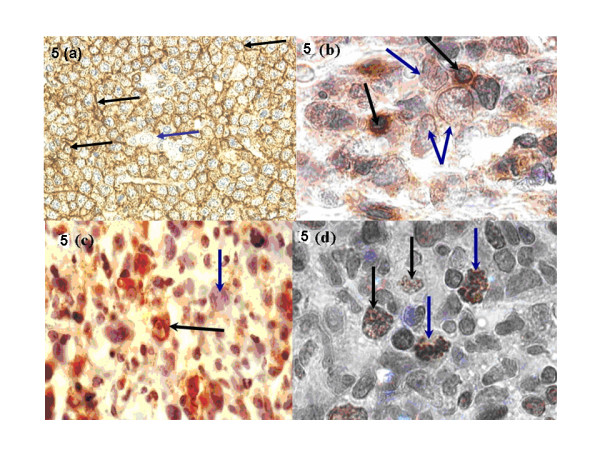
**a) **Anti-CD20 (B-cell) antigen immunoperoxidase reactivity in DLBCL; note the granular cytoplasmic reactivity (brown) in all malignant cells [black arrows] while a macrophage did not stain [blue arrow] (× 200). **(b) **Anti-CD3 (T-cell) antigen immunoperoxidase reactivity in DLBCL; note the brown focal cytoplasmic reactivity in tumor infiltrating T-lymphocytes [black arrows] while the malignant cells were not reactive [blue arrows] (× 400). **(c) **Anti-CD68 antigen immunoperoxidase reactivity in DLBCL; note the brown cytoplasmic reactivity in tumor infiltrating macrophages [black arrow] while malignant cells did not stain [blue arrow] (× 400). **(d) **Anti-Ki-67 (proliferation) antigen immunoperoxidase reactivity in an aggressive lymphoma: note the brown granular nuclear reactivity in proliferating cells [black arrows] including the abnormal mitotic bodies [blue arrows] (× 400).

Immunohistological reactivity for the various antigens studied are summarized in table 4 and illustrated on figures 5(a–d). All stained BL (15/34) and NHL (40/103) cases and some cells in stained HD biopsies (15/39) showed reactivity for leucocyte common antigen (CD45; LCA), and the B-cell antigen (CD20) [Figure 5(a)]. The LCA and CD20 reactivity was localized on cell membranes and diffusely dispersed within the tumour tissue. More than 90% of the tumor cells showed reactivity indicating a lymphocytic B-cell lineage. T-cell (CD3) and macrophage antigens (CD68) [Figure 5(b) &5(c)] were expressed on tumor infiltrating T-cells and macrophages in variable frequency with cases suggesting varying inflammatory/immunological reactivity to the tumor cells. HD cases showed variable immunoreactivity on the cells for LCA and CD20 depending on the histological type but the RS cells were not reactive to CD20. CD3 and CD68 expressions were focal but variable depending on the histological subtype (Table 4). Variable expression of nuclear Ki-67 was seen in lymphomas and included a mean positive cell/HPF of 70.0% for BL, 40.0% for NHL and 25.0% in HD [Figure 5(d)].

**Table 4:** Immunohistochemical characteristics of ML at MNH

	IMMUNOREACTIVITY FOR:				
	
TUMOUR	LCA*	CD20	CD3	CD68	MEAN Ki-67 (% +/HPF)
**BL**	Reactive (membranous cytoplasmic) in all tumour and infiltrating cells	Reactive (membranous cytoplasmic) in all tumour cells	Focal (membranous cytoplasmic) reactivity in infiltrating T-cells	Focal (membranous cytoplasmic) reactivity in infiltrating phagocytes	70.0
**NHL**	Reactive (membranous cytoplasmic) in all tumour and infiltrating cells	Reactive (membranous cytoplasmic) in all tumour cells	Focal (membranous cytoplasmic) reactivity in infiltrating T-cells	Focal (membranous cytoplasmic) reactivity in infiltrating phagocytes	40.0
**HD**	Reactive (membranous cytoplasmic) in all tumour and infiltrating cells	Focal reactivity in infiltrating B-cells while RS and Hodgkin cells negative	Focal reactivity in infiltrating T-cells	Focal (membranous cytoplasmic) reactivity in infiltrating phagocytes	25.0

*LCA: Leucocyte common antigen (CD45)

### Concordance between clinical and histological diagnosis at MNH

A clinical diagnosis of ML was available in only 117 of the 176 (66.5%) cases studied. The overall concordance between the clinical and histological diagnosis of ML was 62.4% (n = 73/117). Unexpectedly, concordance was highest for HD (76.9%, n = 20/26) than BL (47.8%, n = 11/23) although the latter may seem easier to diagnose clinically because of the characteristic age and anatomical presentation but these variations were not statistically significant, p-value = 0.1091.

## Discussion

The combined frequency of major types of ML in the current tumor biopsy study in Tanzania (4–5%) is generally comparable to that reported in developed countries including a range of 2.9–4.9% in the USA and about 3.5% in Europe. [1,23,24] This is interesting considering the fact that an increasingly major influence in African ML pathogenesis is supposedly HIV and AIDS whose epicenter is in sub-Saharan Africa which includes Tanzania. [25,26] Furthermore, racial and geographic differences are also known to contribute to ML distribution globally. [24,27] This global similarity, could however, in part be explained by the ubiquitous distribution of EBV which is also considered a major ML pathogen. [27,28] Although this study is hospital-based allowing an insight into ML frequency at MNH, it is not sufficient to offer a solid basis for comparison with other countries.

The apparent increase in frequency of total ML between 1997 and 2000 appears largely to be contributed by NHL as well as HD but not BL which is in agreement with our previous report of increasing ML frequency in the early 1990's (2^nd ^post-HIV) decade [12] and indicating a role of the HIV and AIDS epidemic as also reported elsewhere. [12] That NHL had a leading frequency followed by HD and BL is largely in agreement with reports from other studies including a previous Tanzanian report [29] and an East Malaysian study. [30] Conversely, an MNH study from the pre-HIV epidemic era of a cervical lymphadenopathy pediatric series indicated a higher (34.5%) frequency of HD relative to NHL. [31] These studies together support the notion that HIV and AIDS influence the frequency and distribution of lymphomas worldwide including the fact that some ML are strongly associated with AIDS and/or may be AIDS-defining. [2,6,12] These results however, are not inline with the low (≈20%) HIV frequency observed in the current lymphoma cohort due to the rather sparse HIV screening of ML at MNH which does not allow definitive evaluation of ARL in Tanzania. Despite this fact however, it is noteworthy that a slight majority (55.6%) of NHL and a significant proportion (44.4%) of HD tested were HIV seropositive suggesting an increased ML association with the AIDS epidemic among the biopsies submitted at the hospital during the study period.

The male predominance for ML in this study, compares more with a Pakistan study (ratio 1.9:1) [32] than with a report from neighboring Kenya (ratio 1.2:1) which is surprising. [33] However, the reasons for a lower M:F ratio in Kenya than in Tanzania are not clear but they may include differences in hospital seeking behavior or under-reporting in Kenya. Considering that all ML subtypes showed male preponderance it is not clear whether differences in sex, biological pathogenesis and/or yet unclear socio-environmental factors influence this distribution although a selection bias and differences in hospital seeking behavior between sexes could also be considered.

The age distribution of the studied Tanzanian ML cohort as well as of the NHL cases appears similar to that reported in studies from neighboring Kenya and Uganda as well as Pakistan. [32-34] Likewise, the peak age for BL patients in our study is concordant to that reported in Kenya [15] and elsewhere. [14,20] Unexpectedly however, HD cases in our current study did not show the usual clear bimodal age pattern although the mean age of 26 years was concordant with that reported in a previous Ugandan study. [34] Apparently, the mean age in our series and the Ugandan study indicate that East African HD presents at a considerably younger age than that reported in USA (≈33 years) and Taiwan (41.5 years) [13,34,35] The reasons for the differences are not clear although these may include age-related oncogenic factors, geographical and other environmental factors as well as the influence of HIV infection in sub-Saharan Africa, considering the above-stated proportion of HIV infected HD patients at MNH in our current study. [24] Reports show that childhood HD occurs at a younger age in various countries with limited resources than in western countries. Thus as many as 20 to 30% of childhood HD cases in developing countries occur before 5 years of age against some 5% in industrialised countries. [36,37] Furthermore, it is reported that there is an inverse relationship between the incidence of the HD in children and young adults within countries according to their economic development. [37] Low socio-economic status may generate malnutrition, which could lead to impaired immunity after initial exposure, and so to development of HD. [37]

The HGNHL (B-cell type) association with HIV in the current study was expected and is consistent with previous reports. [9,33] However, the well known ARL including PEL and PCNSL were not found in the present cohort probably due to inadequate clinical surveillance for them (including pleural biopsies and cerebro-spinal fluid (CSF) cytology). Our finding that MC was the most frequent HD subtype is consistent with other reports. [38] Furthermore, the MC subtype has also been found to be most frequent in the setting of HIV and AIDS. [11,13] Considered together these reports and our current study suggest that, the presentation of HIV-associated HD is different from that which is not HIV associated. [6] An association between BL and HIV could not be established in the current study due to rather small numbers of tested patients who were all seronegative. However, BL has been shown to be associated with HIV in other studies. [39,40] Noteworthy also, was that most NHL seropositive for HIV in the current study presented with generalized lymphadenopathy, apparently indicating a more aggressive clinical course as compared to the non-HIV associated ML. Recent studies have also reported a correlation between HIV infection and disseminated ML with lymphadenopathy, scalp and bone marrow involvement (anatomical distribution) and pancytopenia. [33,41]

The Ki-67 immunoreactivity (cell proliferation) seen among BL, NHL and HD cases at MNH is comparable with that in other reports [42,43] but the value of such proliferation markers like Ki-67 and cyclin-A (Cy-A) as prognostic indicators in Tanzania needs extended studies.

Although reasonable concordance was observed between clinical and histopathological diagnosis of ML at MNH, further diagnostic improvements are needed to allow an international comparison.

## Conclusion

Lymphomas apparently increased among the number of tumors diagnosed at MNH between 1996 and 2001 frequently appearing related to the HIV epidemic, often presenting early and peaking in young age-groups. The frequent aggressive clinical and histological presentation as well as the dominant B-immunophenotype appears also to suggest an association with HIV and AIDS. Thus, routine HIV screening of all malignant lymphoma patients at MNH is necessary in order to allow comprehensive ARL determination and formulation of preventive and therapeutic protocols.

## Competing interests

The authors declare that they have no competing interests.

## Authors' contributions

ARM conceived the study, collected tissue specimen, clinical records, performed the staining, performed microphotography, data analysis and manuscript writing, EEK conceived and supervised the study, procured reagents, taught and supervised the staining, microphotography and corrected the manuscript, EMM supervised proposal write-up, data analysis and corrected the manuscript.
